# The Effects of Anthropogenic Structures on Habitat Connectivity and the Potential Spread of Non-Native Invertebrate Species in the Offshore Environment

**DOI:** 10.1371/journal.pone.0152261

**Published:** 2016-03-31

**Authors:** Rachel D. Simons, Henry M. Page, Susan Zaleski, Robert Miller, Jenifer E. Dugan, Donna M. Schroeder, Brandon Doheny

**Affiliations:** 1 Earth Research Institute, University of California Santa Barbara, Santa Barbara, California, United States of America; 2 Marine Science Institute, University of California Santa Barbara, Santa Barbara, California, United States of America; 3 Bureau of Ocean Energy Management, Pacific Region, Camarillo, California, United States of America; University of Sydney, AUSTRALIA

## Abstract

Offshore structures provide habitat that could facilitate species range expansions and the introduction of non-native species into new geographic areas. Surveys of assemblages of seven offshore oil and gas platforms in the Santa Barbara Channel revealed a change in distribution of the non-native sessile invertebrate *Watersipora subtorquata*, a bryozoan with a planktonic larval duration (PLD) of 24 hours or less, from one platform in 2001 to four platforms in 2013. We use a three-dimensional biophysical model to assess whether larval dispersal via currents from harbors to platforms and among platforms is a plausible mechanism to explain the change in distribution of *Watersipora* and to predict potential spread to other platforms in the future. Hull fouling is another possible mechanism to explain the change in distribution of *Watersipora*. We find that larval dispersal via currents could account for the increase in distribution of *Watersipora* from one to four platforms and that *Watersipora* is unlikely to spread from these four platforms to additional platforms through larval dispersal. Our results also suggest that larvae with PLDs of 24 hours or less released from offshore platforms can attain much greater dispersal distances than larvae with PLDs of 24 hours or less released from nearshore habitat. We hypothesize that the enhanced dispersal distance of larvae released from offshore platforms is driven by a combination of the offshore hydrodynamic environment, larval behavior, and larval release above the seafloor.

## Introduction

Connectivity of habitats through the dispersal of reproductive propagules, such as seeds, spores, and larvae, is a major driver of population dynamics, community structure, gene flow, and the distribution of native and non-native species in terrestrial and marine ecosystems [1–5]. For the majority of marine invertebrate species, the principal dispersal stage is a planktonic larva. Connectivity among populations and habitats is related to the duration of this planktonic stage and to physical and biological factors that affect larval transport and survival [2, 6, 7]. Human-mediated activities in the marine environment can increase larval connectivity and introduce non-native species to new habitats. The transport of non-native species as larvae in ballast water or as adults attached to boat hulls are often cited examples of human facilitated dispersal of non-native species to new regions [8–10].

It has been suggested that offshore energy structures, such as oil and gas platforms [3, 11] and wind farms [12], can facilitate species range expansions and the introduction of non-native species into new geographic areas. These structures are often situated in a soft seafloor environment, providing vertical and shaded hard substrate habitat where it would not normally exist. As a result, these structures provide patches of habitat or “stepping stones” that could facilitate the dispersal of species into new areas [3, 11–13]. Such effects are likely to vary with physical and biological factors that include proximity to inshore habitat that could act as a source of propagules, the number and spacing of structures, local and regional current patterns, and species life histories [12]. However, few studies have explicitly explored potential larval connectivity among existing offshore structures or their possible role in the dispersal of non-native species despite the need for such information [14–16].

Potential connectivity among offshore platforms can be explored using biophysical models of larval dispersal [2, 17–19]. We define potential connectivity as the probability of larval transport from a source site to a destination site via currents [20, 21]. Biophysical models have been widely used to investigate dispersal patterns and connectivity among habitats for invertebrates and fish with planktonic larval durations (PLDs) ranging from days to months [17]. However, larval dispersal of marine invertebrates with PLDs of 24 hours or less has been rarely investigated using biophysical models. Limited field studies have suggested that the larval dispersal distances of species with PLDs of 24 hours or less are on the order of meters to 100s of meters [22, 23], which may be one reason why connectivity modeling of these species is uncommon.

**Fig 1:**
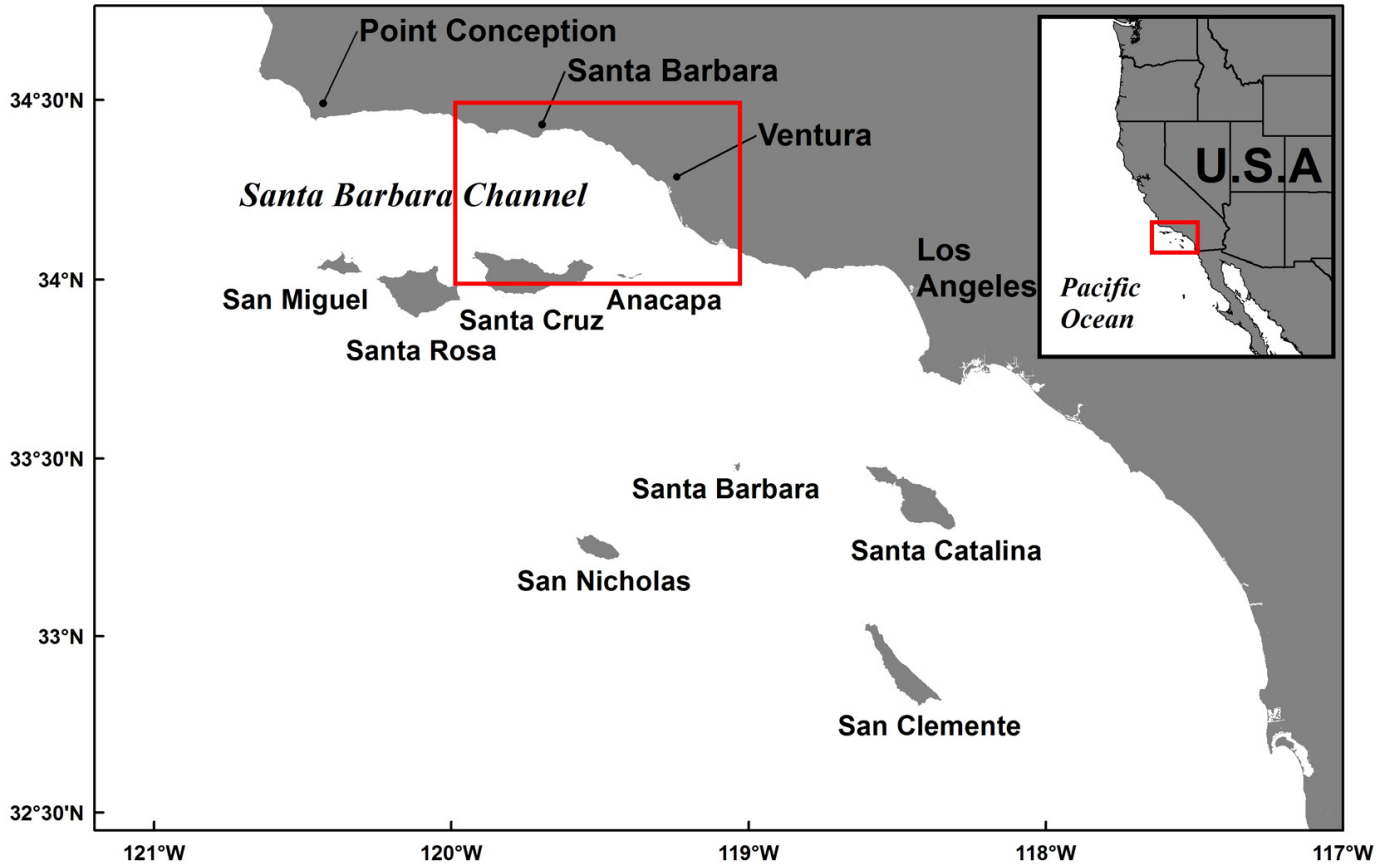
Southern California Bight and model domain. The study area, shown by the red box, is located in the eastern Santa Barbara Channel.

Surveys of sessile invertebrates on seven offshore oil and gas platforms in the Santa Barbara Channel (SBC, Fig 1) in 2001 revealed the non-native encrusting bryozoan *Watersipora subtorquata* (= *W*. *subatra*?, [24], hereafter *Watersipora*) on one of the seven platforms [25]. *Watersipora* is now common in the harbors and coastal embayments of central and southern California [26], but rarely reported in more open coastal habitat [25]. Under favorable conditions, *Watersipora* is an aggressive competitor for space [27], overgrowing and excluding other benthic epifauna during growth (e.g. barnacles and bivalves) and acting as a foundation species or a “bioengineer” by forming large (several decimeter to larger) three-dimensional masses that provide a novel habitat for invertebrate taxa [27, 28]. *Watersipora* has short lecithotropic larval stage with an estimated maximum PLD of 24 hours [29, 30].

**Fig 2:**
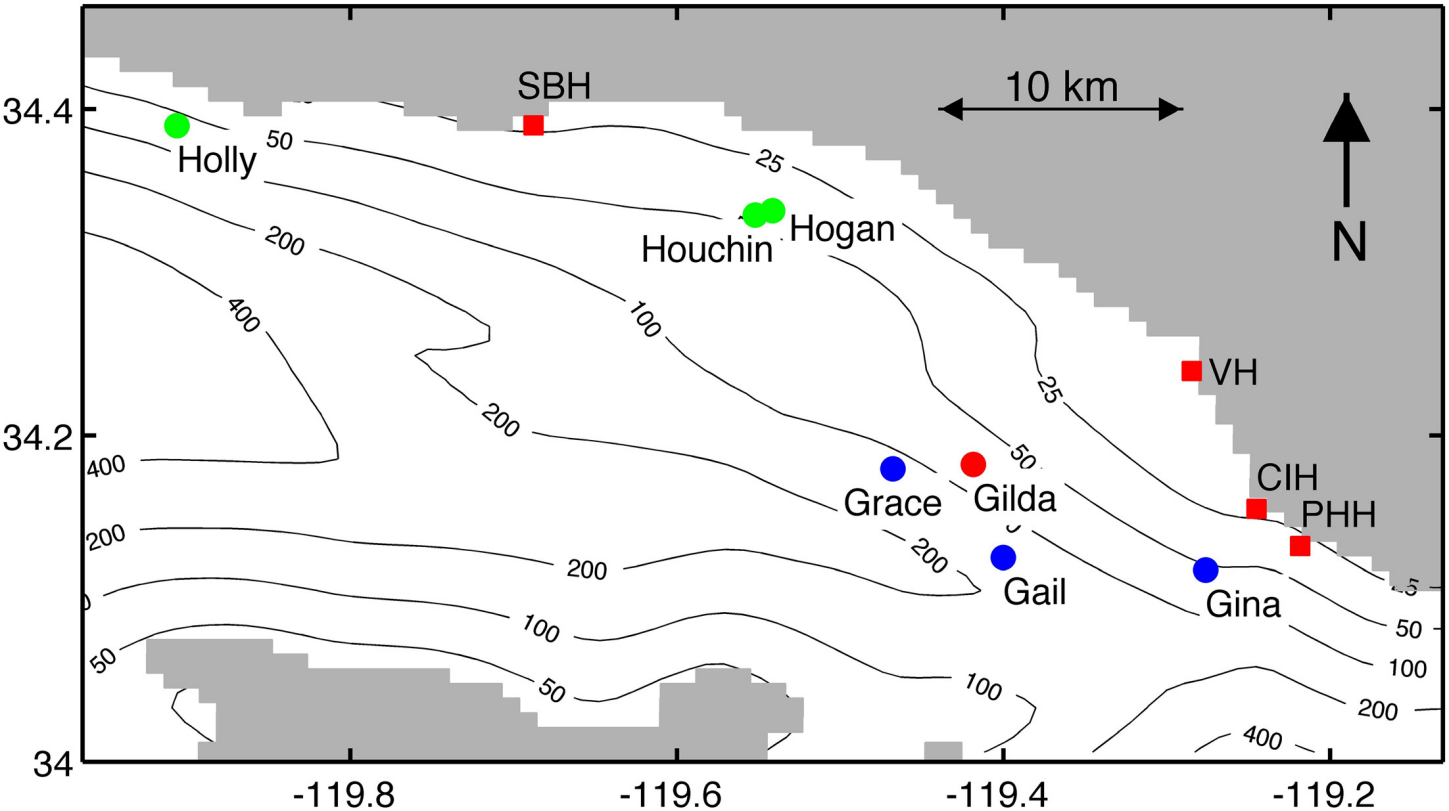
Locations of oil and gas platforms (circles) and harbors (squares). Red symbols identify the locations where *Watersipora* was assumed present or observed in 2001 and 2013. Blue symbols identify the locations where *Watersipora* was present in 2013, but not in 2001. Green symbols identify the locations where *Watersipora* was not present in 2001 or 2013. SBH = Santa Barbara Harbor, VH = Ventura Harbor, CIH = Channel Islands Harbor, and PHH = Port Hueneme Harbor. Bathymetry contours in meters are shown by the black lines.

In this study, we investigate the potential connectivity of *Watersipora* between seven offshore oil and gas platforms in the SBC (Fig 2). The transport and connectivity of *Watersipora* larvae is estimated using a three-dimensional biophysical model, which consists of an ocean circulation model to simulate flow and a particle tracking model to simulate larval transport. We use the biophysical model to assess whether habitat connectivity via larval dispersal is a plausible mechanism to explain an observed spread of *Watersipora* from one platform in 2001 to four platforms in 2013. Hull fouling is also a possible mechanism for the dispersal of *Watersipora* among habitats [31, 32]. *Watersipora* is widely distributed in the harbors of southern California and has been documented in the four harbors, Santa Barbara, Ventura, Channel Islands, and Port Hueneme, inshore of our study platforms [29, 33–36]. Thus, these four harbors are included as potential sources of *Watersipora* larvae in our modeling. Our study also examines the influence of the offshore hydrodynamic environment on larval dispersal distances for taxa with PLDs of 24 hours or less.

## Methods

### Distribution and abundance of *Watersipora* on platforms

We documented changes in the distribution and abundance of *Watersipora* from 2001 to 2013 using SCUBA surveys of seven offshore oil and gas platforms located in the western SBC (Fig 2). The following companies issued permission to dive the platforms: Veneco, Inc. (platforms Holly, Grace and Gail), Pacific Operators Offshore, LLC. (platforms Houchin and Hogan), and Nuevo Energy in 2001 and DCOR, LLC. in 2013 (platforms Gilda and Gina). The study platforms encompassed a range of sizes, water depths, and distances from shore (Table 1, Fig 2, [25, 37]). The submerged portion of the platforms consisted of vertical, oblique, and horizontal cylindrical steel support members and vertical conductor pipes through which the wells are drilled. The hard substrate provided by the submerged structure was typically occupied subtidally by a diverse assemblage of sessile and semi-mobile invertebrates, including mussels (*Mytilus californianus*, *M*. *galloprovincialis*), barnacles (e.g. *Megabalanus californicus*), rock scallops (*Crassodoma gigantea*,), and anemones (*Corynactis californica*, *Metridium senile*) [37, 38]. The support structures and conductor pipes of the platforms are cleaned infrequently, usually years apart, and typically to a depth of ~9 m. *Watersipora* colonies are negatively buoyant and when dislodged from the platforms, the fragments drop to the seafloor (diver observations). Thus, platform cleaning is an unlikely to provide a transmission pathway for the spread of *Watersipora*.

**Table 1:** Characteristics of study platforms.

Variable	Platforms						
	Gina	Gail	Gilda	Grace	Hogan	Houchin	Holly
Year of Installation	1980	1987	1981	1979	1967	1968	1966
Distance from shore (km)	5.0	13.2	11.9	14.4	5.1	7.0	2.9
Water depth (m)	29	225	64	97	46	49	64
Platform size (m^2^ on bottom)	560	5,600	2,340	3,120	1,444	1,444	1,728

To measure the distribution and abundance of *Watersipora*, we used a camera enclosed in an underwater housing with two strobes mounted on a quadrapod designed to photograph 0.25 m^2^ plots following methods modified from Coyer et al. [39]. Plots measured 41 cm x 62 cm to accommodate the dimensions of the platform legs and conductor pipes. We photographed one 0.25 m^2^ plot on the inside and outside of each of the four corner legs and four randomly selected conductor pipes at depths of 6 m, 12 m, and 18 m for a total of 48 photoplots per platform. Additional qualitative swimming surveys of approximately 30 minutes were done among the conductor pipes at each depth searching for presence of *Watersipora*. Surveys were weather and access dependent and conducted from late August to early November in 2001 and 2013. The time to survey a particular platform varied between 1–2 days depending on platform size.

We identified and estimated the percentage cover of *Watersipora* within each photoplot using point-contact methods. The image from each photoplot was projected onto 100 uniformly distributed points and points with *Watersipora*, contacts, were recorded to estimate cover. The same plot locations were surveyed in 2001 and 2013. We also consulted previous survey data of some of the platforms conducted by others in October 1999 and 2000 for records of *Watersipora* [40].

### Biophysical modeling of larval dispersal and connectivity

A three-dimensional biophysical model was used to estimate larval dispersal of *Watersipora* from the seven oil and gas platforms and four harbors in our study area (Fig 2). The biophysical model combined an ocean circulation model and a particle tracking model, where the particles represent simulated larvae. The three-dimensional ocean circulation model was a high-resolution Regional Ocean Modeling System (ROMS) applied to the Southern California Bight [41, 42]. The model domain covered the southern California coastline including the eight Channel Islands (Fig 1). The model grid was 258 km by 386 km with a 1 km horizontal resolution and 40 vertical levels. Detailed information on the lateral and surface boundary conditions and model validation can be found in Dong and McWilliams [41] and Dong et al. [43]. The model has been rigorously calibrated against field observations and shown to accurately capture mean, interannual, seasonal, and intraseasonal mesoscale dynamics of the Southern California Bight, which includes the SBC [43–45]. Thus, the model resolution is adequate to estimate larval dispersal distances of 1 km or larger. The three-dimensional particle tracking model was driven by 6-hour averaged three-dimensional velocity fields produced by the ROMS following the methods in Mitarai et al. [20] and Carr et al. [46]. For this study, the ROMS velocity fields were available for 12 years from 1996–2007. Particles were moved forward in time using a fourth-order accurate Adams-Bashforth-Moulton predictor-corrector scheme and a 900 s time step. The particle tracking model was validated against observational data from drifter experiments by Ohlmann and Mitarai [47].

To model the potential connectivity among the platforms and harbors, particles were released from eight source sites in the study area; platforms Grace, Gilda, Gail, and Gina and Santa Barbara, Ventura, Channel Islands, and Port Hueneme harbors (Fig 2). Following Watson et al. [21] and Mitarai et al. [20], potential connectivity is defined as the probability of larval transport from a source to a destination location as estimated by particle tracking simulations. In the absence of data on larval production and survivorship, estimates of potential connectivity were best suited to this study. As the model grid was 1 km^2^ in the horizontal direction, the details of the harbor bathymetry could not be included in the model. To release particles from the harbors, the particles were placed at the first water grid cell adjacent to the harbor location near the shoreline. This procedure assumes that *Watersipora* larvae can be transported out of the harbor by ebbing tidal currents and into open water, which is supported by the presence of *Watersipora* on a wharf near the entrance to Santa Barbara harbor (personal observation). At each source site, particles were released vertically every 0.1 meters from 1 to 18 meters below the surface, the depth range at which *Watersipora* colonies were observed at the platforms [25]. Particles were released every 3 hours and tracked passively for 24 hours, based on the estimated maximum PLD of *Watersipora* [29, 30]. Typical of other bryozoan taxa, *Watersipora* larvae are small and weak swimmers [48, 49]. *Watersipora* larvae initially show positive phototaxis on release ([50], personal observation), but due to their size and weak swimming, it is unlikely that they could change their vertical position in the water column to influence their horizontal transport. Due to *Watersipora*’s weak swimming ability and short PLD along with the strong offshore horizontal currents in the SBC, larvae can be realistically modeled as passive particles. To address any potential variability in the depth distribution of larvae in the water column, particles were released over the top 18 m of the water column where *Watersipora* was observed on the platforms. The particle release frequency was selected to meet the criteria for robustness in particle tracking models [51]. Particles were released for June through August, the estimated reproductive season for *Watersipora* (unpublished data, see S1 Table), for 12 years from 1996 to 2007. For this study, approximately seven million particle trajectories were simulated with 875,000 particles released from each of the eight sources. The number of particles was selected to achieve model robustness following the methods in Simons et al. [51].

To estimate the extent of larval dispersal, the individual particle trajectories, calculated by the biophysical model, were transformed into two-dimensional particle density distributions (PDDs) for each source site. Since *Watersipora* larvae were assumed to have a PLD of 3–24 hours [26, 30], the particle locations from each trajectory were saved every 3 hours up to 24 hours after their release. Using the three-dimensional distribution of all particles released from a platform or harbor over the reproductive season of June to August for a single year, an annual PDD was produced by summing the number of particles within a grid cell over depth and then dividing by the total number of particles released [20]. The annual PDDs for each source were then averaged over the 12 model years from 1996–2007 to obtain a long-term average of particle dispersal. Although the model years of 1996–2007 did not coincide exactly with the years between the surveys of 2001–2013, the model provided a long-term average of particle dispersal, which was applicable to the 12-year period between the surveys. By sampling the values of the 12-year averaged PDDs from the source platforms and harbors at the seven destination platforms, potential connectivity was quantified in the form of a matrix. The values of the connectivity matrix represent the fraction of the total number of particles released from a source site that arrived at a destination site, which can be converted to a percentage by multiplying the matrix by 100. Overall, the connectivity matrix illustrates the relative degree of potential connectivity between the source and destination sites.

## Results

### Distribution and abundance of *Watersipora* on platforms

**Fig 3:**
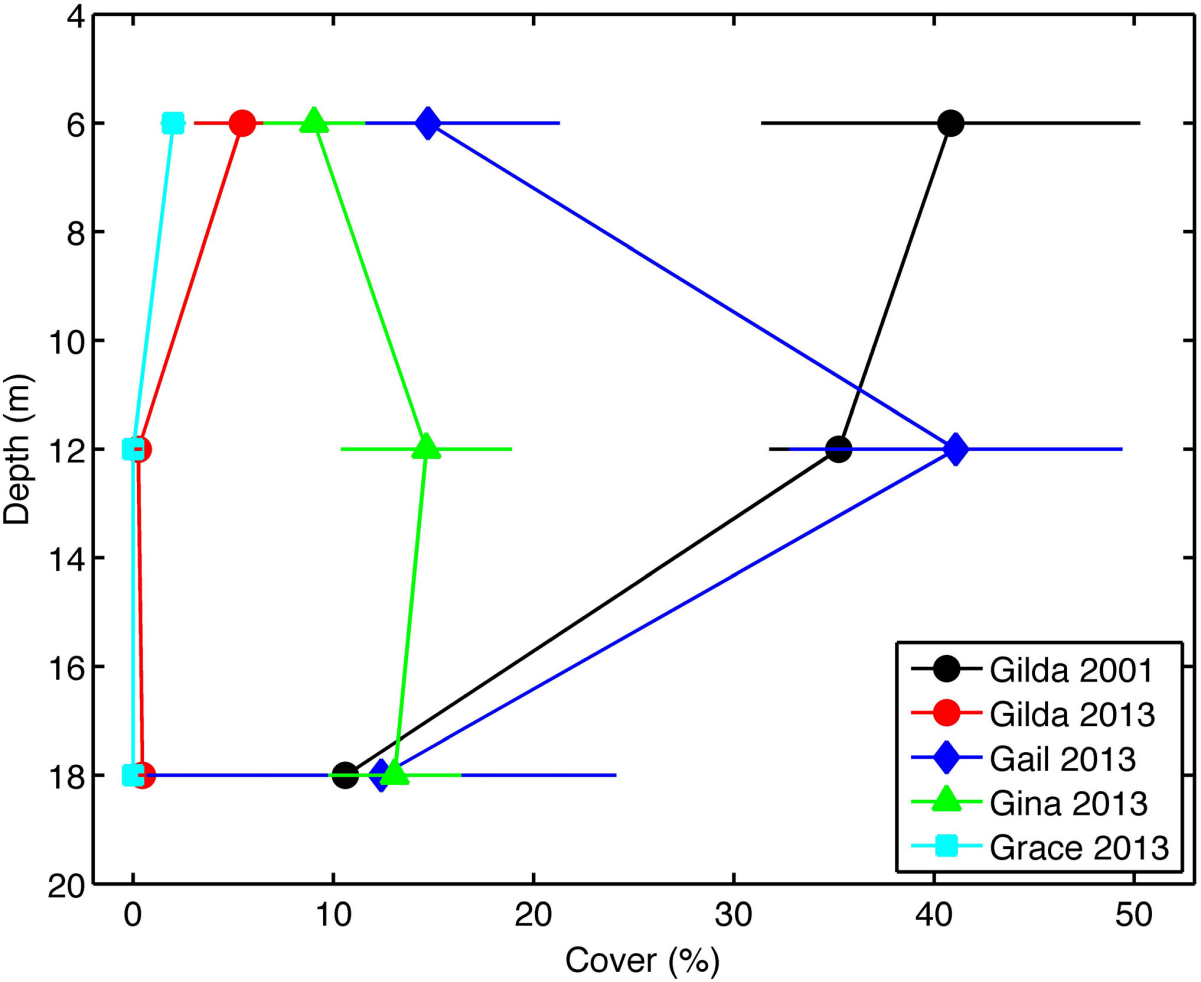
Percent cover of *Watersipora* at depths of 6 m, 12 m, and 18 m on platform Gilda in 2001 and 2013 and on platforms Gail, Gina, and Grace in 2013. *Watersipora* was absent from platforms Gail, Gina, and Grace in 2001. The percent cover is displayed as mean values ± one standard error.

Our 2001 surveys revealed *Watersipora* on only one of the seven study platforms, platform Gilda (Fig 3) [25]. At platform Gilda in 2001, the mean cover of *Watersipora* decreased with depth from 40.8% ± 9.5% SE at 6 m to 10.6% ± 3.7% SE at 18 m. An independent survey of study platforms Gail and Grace in 1998–2000 using SCUBA divers and remotely operated vehicles also failed to find *Watersipora* [40]. Our 2013 surveys found that the distribution of *Watersipora* had expanded to include 3 additional platforms, Grace, Gail and Gina, with the cover of *Watersipora* varying among platforms and depths (Fig 3). The highest mean percent cover occurred on platform Gail (41.1% ± 8.3% SE) at the intermediate depth of 12 m. The mean percent cover was lowest (2.0% ± 0.6% SE) on platform Grace, where only small colonies were found at a depth of 6 m. Platform Gilda, the site of the first record of *Watersipora* on a platform in 2001, had been recently cleaned with the invertebrate assemblage removed to a depth of approximately12 m and mean coverage at all depths had decreased to less than 6%.

### Biophysical modeling of larval dispersal and connectivity

Based on our survey results, two modeling scenarios were used to explore the potential dispersal and connectivity of *Watersipora* larvae among seven platforms and four harbors in the SBC. In the first scenario, particles were released from platform Gilda, where *Watersipora* was observed in 2001, and the four harbors and tracked to all seven platforms. In the second scenario, particles were released from platforms Grace, Gilda, Gail, and Gina, where *Watersipora* was observed in 2013, and the four harbors and tracked to all seven platforms.

**Fig 4:**
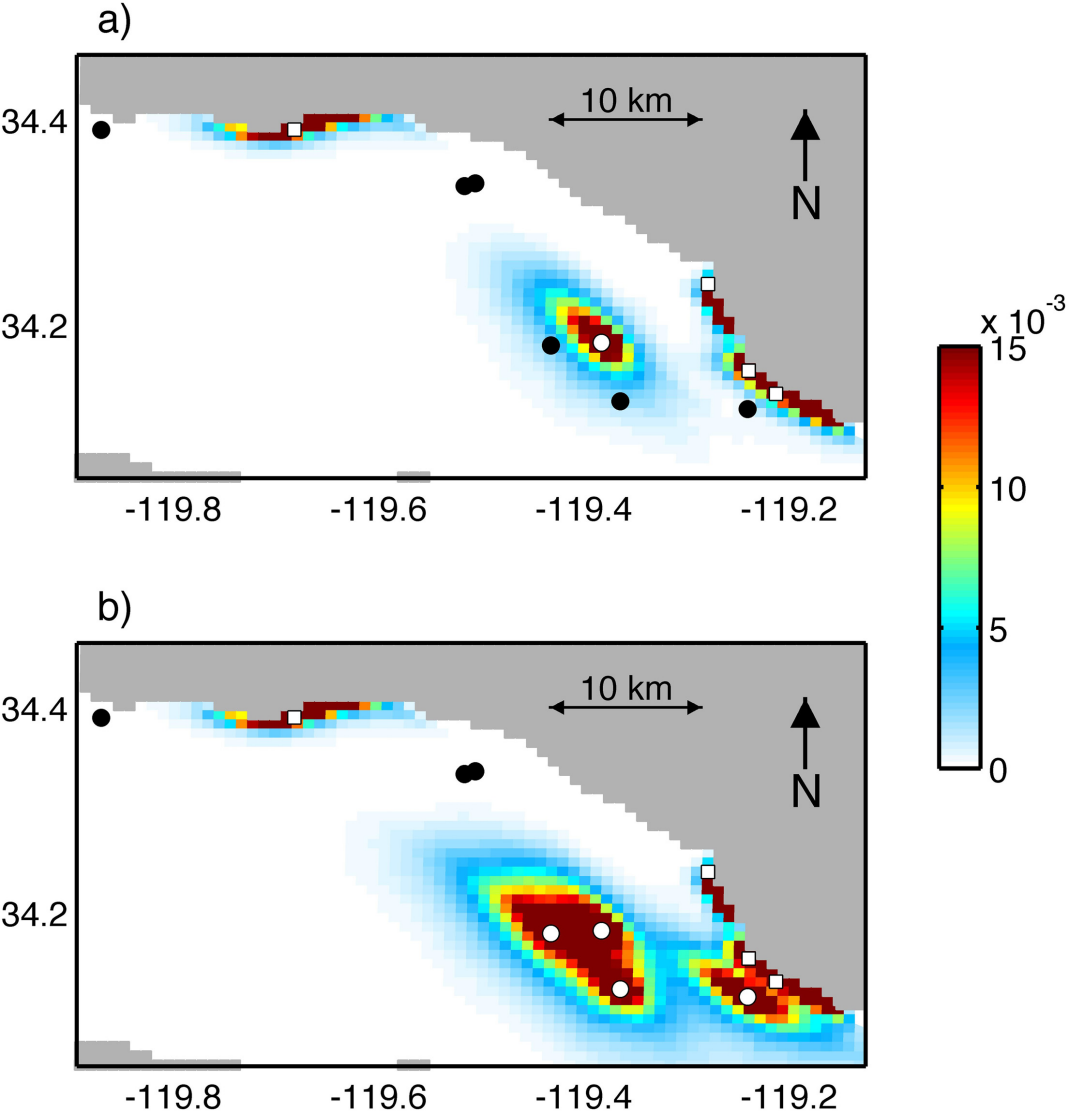
(a) PDDs averaged over 12 years for scenario 1. (b) PDDs averaged over 12 years for scenario 2. White circles and squares identify the platforms and harbors respectively that are source sites, where particles are released. Black circles identify the platforms that are used only as destination sites.

In order to display the horizontal extent of larval dispersal, the PDDs from the individual source sites for each scenario are added together and displayed in Fig 4. In scenario 1, the particles released from platform Gilda disperse significantly farther than the particles released from the four harbors. Platform Gilda is centrally located in the SBC (Fig 2) and is thus exposed to higher flow than the harbor mouths, which are located near the shoreline. As strong currents run along the basin of the eastern SBC [52], the major axis of the elliptical PDD for platform Gilda aligns with these flows as well as the bathymetric contours shown in Fig 2. The PDD from platform Gilda extends to nearby platforms Grace and Gail, indicating that particles released from platform Gilda can reach these platforms in 24 hours or less. The PDDs from Channel Islands and Port Hueneme harbors also extend to platform Gina, but not to the other six platforms. The PDDs from Ventura and Santa Barbara harbors do no extend to any of the seven platforms. In scenario 2, the PDDs from platforms Gilda, Grace, and Gail overlap such that they are not distinguishable, indicating potential connectivity between these three platforms. The overlapping PDDs from platform Gina, Channel Islands harbor and Port Hueneme harbor suggest potential connectivity between these sources as well. The three northwestern platforms, Holly, Houchin and Hogan, do not display potential connectivity with any of the eight sources in scenario 1 or 2.

For scenario 1, the potential connectivity matrix (Table 2) reveals the highest connectivity from platform Gilda to itself. This self-connectivity indicates high local retention at platform Gilda, which is not unexpected given the short PLD of *Watersipora*. The second highest potential connectivity values in Table 2, on the order of 10^−1^, are from platform Gilda to platforms Grace and Gail, which are 5 km and 7 km respectively from platform Gilda. The potential connectivity matrix also reveals connectivity from Channel Islands and Port Hueneme harbors to platform Gina. Little or no potential connectivity is detected from platform Gilda to the three northwest platforms, Holly, Houchin, and Hogan, or from Ventura or Santa Barbara harbors to any of the seven surveyed platforms.

**Table 2:** Potential connectivity matrix for scenario 1.

Source Sites	Destination Sites						
	Platform Holly	Platform Houchin	Platform Hogan	Platform Grace	Platform Gilda	Platform Gail	Platform Gina
Platform Gilda	0	6.8x10^-4^	0	2.3x10^-1^	4.7	1.7x10^-1^	3.3x10^-3^
Port Hueneme Harbor	0	0	0	0	0	0	2.3x10^-2^
Channel Islands Harbor	0	0	0	0	0	0	2.6x10^-2^
Ventura Harbor	0	0	0	0	0	0	0
Santa Barbara Harbor	2.1x10^-3^	1.4x10^-3^	7.7x10^-4^	0	0	0	0

The values represent the percentage of the total number of particles released from a source site that arrived at a destination site.

For scenario 2, the potential connectivity matrix (Table 3) reveals self-connectivity or high local retention at each of the four source platforms, Grace, Gilda, Gail, and Gina. High potential connectivity is also predicted between platforms Grace, Gilda, and Gail as indicated by the second highest values in Table 3, on the order of 10^−1^. In addition to Channel Islands and Port Hueneme harbors, platform Gina now shows a similar level of potential connectivity with platform Gail. Even with the additional sources of platforms Grace, Gail, and Gina, the platforms to the northwest, Holly, Houchin, and Hogan, continue to show little to no potential connectivity with the platforms to the southeast, Grace, Gilda, Gail, and Gina.

**Table 3:** Potential connectivity matrix for scenario 2.

Source Sites	Destination Sites						
	Platform Holly	Platform Houchin	Platform Hogan	Platform Grace	Platform Gilda	Platform Gail	Platform Gina
Platform Gina	0	0	0	1.9x10^-3^	2.1x10^-2^	1.7x10^-2^	4.6
Platform Gail	0	0	0	4.1x10^-1^	1.2x10^-1^	2.8	1.0x10^-2^
Platform Gilda	0	6.8x10^-4^	0	2.3x10^-1^	4.7	1.7x10^-1^	3.3x10^-3^
Platform Grace	0	0	0	2.9	1.0x10^-1^	8.1x10^-2^	1.4x10^-3^
Port Hueneme Harbor	0	0	0	0	0	0	2.3x10^-2^
Channel Islands Harbor	0	0	0	0	0	0	2.6x10^-2^
Ventura Harbor	0	0	0	0	0	0	0
Santa Barbara Harbor	2.1x10^-3^	1.4x10^-3^	7.7x10^-4^	0	0	0	0

The values represent the percentage of the total number of particles released from a source site that arrived at a destination site.

**Fig 5:**
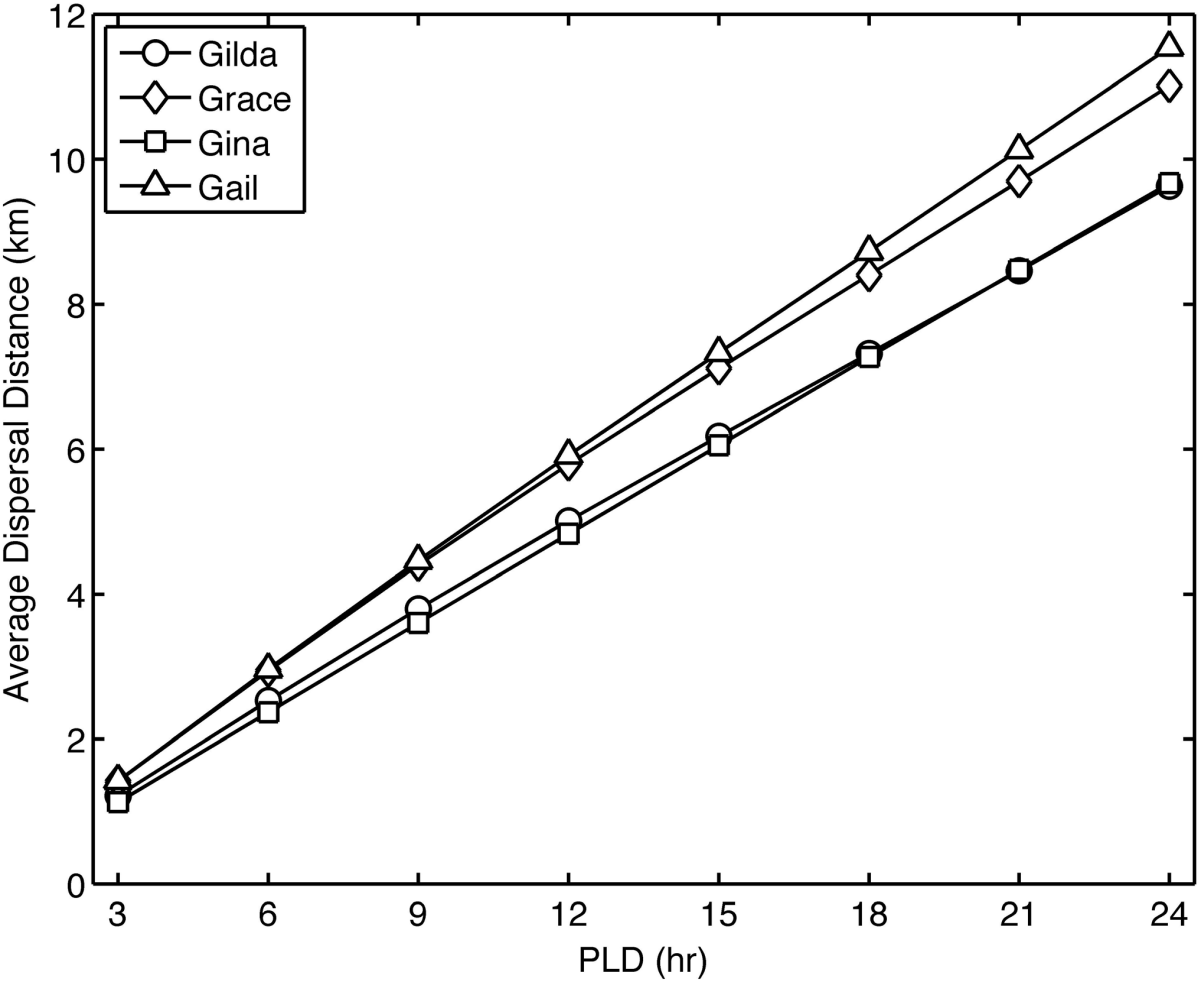
Average dispersal distance of particles (km) versus PLD (hr) for platforms Gilda, Grace, Gina, and Gail. PLD equates to the travel time of the particles.

By calculating the average distance traveled by the particles released from platforms Gilda, Grace, Gina, and Gail over the 12 model years, we explore the relationship between the range of PLDs used for *Watersipora*, 3–24 hours, and the average dispersal distance traveled by the particles (Fig 5). In Fig 5, the PLD equates to the travel time of the particles. For all four platforms, the average dispersal distance increases linearly with increasing PLD. The average dispersal distances for the four platforms range from 1.1 to 1.4 km at a PLD of 3 hours and from 9.6 to 11.5 km at a PLD of 24 hours. In Fig 5, the average dispersal distance is greater for platforms Gail and Grace than platforms Gilda and Gina. Platforms Gail and Grace are located farther offshore in deeper water than platforms Gilda and Gina (Fig 2) and are thus exposed to higher flows, driving a greater dispersal distance.

## Discussion

### Larval connectivity between platforms and harbors

Our modeling study explores whether larval dispersal needs to be considered, along with hull fouling, as a potential pathway for the spread of *Watersipora* among platforms and harbors in the SBC. Estimates of no potential connectivity from the harbors to platform Gilda shown in Table 2 suggest that the colonization of platform Gilda by *Watersipora* prior to 2001 was not due to larval dispersal from the four harbors. Thus, we hypothesize that hull fouling was most likely the initial vector of introduction at platform Gilda. Hull fouling could have occurred via service vessel traffic, including crew boats and barges, or less likely from recreational boats, which are not permitted to tie up or closely approach offshore platforms in the SBC. Multiple commercial boat companies service the platforms in the SBC. One boat company usually provides services to one or two oil companies with specific boats dedicated to specific platforms or sets of platforms owned by the same oil company. However, we have no information on whether *Watersipora* was attached to the boat hulls or on the past frequency and pathways of boat traffic and are thus unable to quantify this potential transmission pathway. Since the link between boat traffic and the spread of *Watersipora* in the SBC remains ambiguous, hull fouling must be considered a potential transmission pathway to explain the spread of *Watersipora* in the SBC.

**Fig 6:**
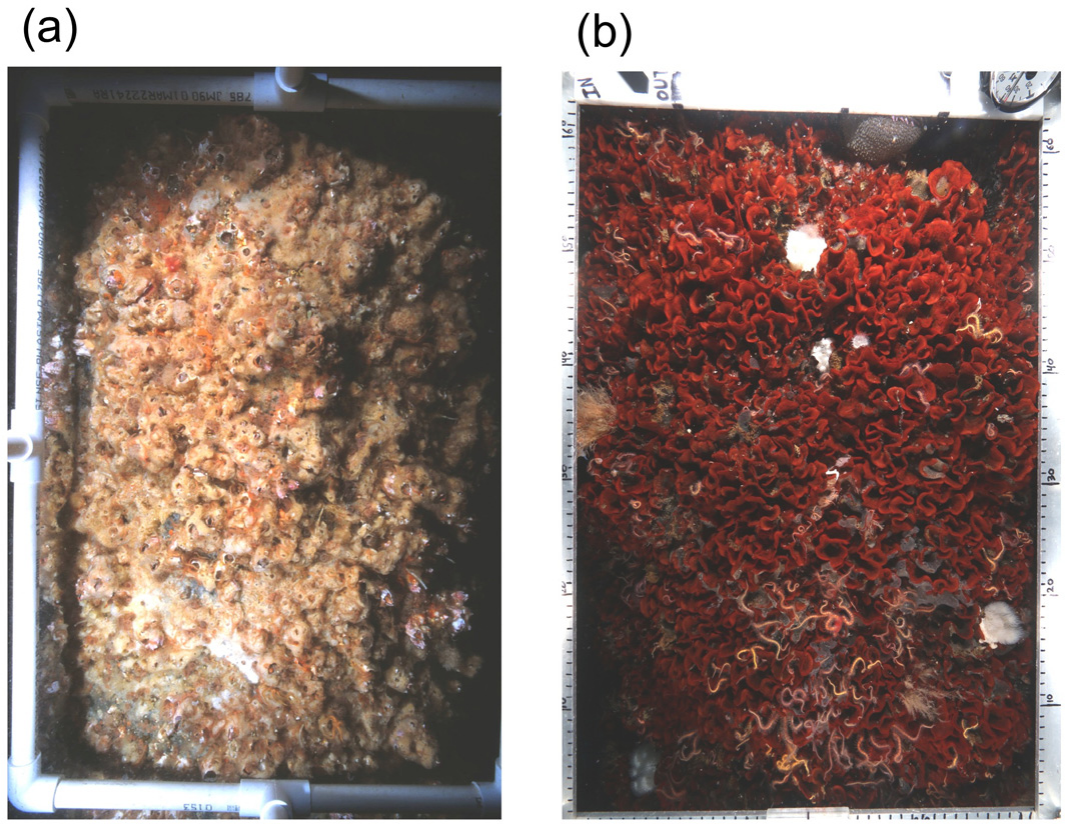
Photographs of the same sample plot on platform Gail (a) in 2001 with plot dominated by barnacles and (b) in 2013 with plot dominated by *Watersipora*. Sample plot was located on a conductor pipe at 9 m depth and measured 41 x 62 cm internal diameter (0.25 m^2^).

Our modeling results reveal three distinct patterns of larval dispersal and potential connectivity among platforms and harbors in the SBC. First, the modeling estimates the highest potential connectivity among the four southeastern platforms, Grace, Gilda, Gail, and Gina. These results are consistent with field surveys from 2001 and 2013, which revealed the spread of *Watersipora* from a single platform, Gilda, to three previously uninvaded platforms, Grace, Gail and Gina. Due to *Watersipora*’s short PLD, estimated to be at most 24 hours [8, 30, 33, 37], the modeling predicts high local retention of *Watersipora* larvae within the vicinity of colonized platforms, which is also consistent with our survey results as *Watersipora* was found on platform Gilda in 2001 and 2013. Although our surveys indicated that the cover of *Watersipora* on platform Gilda varied between 2001 and 2013, this taxon is recognized as potentially dominant species, capable of monopolizing space once established through the lateral growth of colonies and the high local retention of short-lived larvae [27, 35]. Thus, it is extremely likely that *Watersipora* remained on platform Gilda during the 12 year period between surveys. To illustrate the aggressive nature of *Watersipora* colonization on the platforms, photographs of the same sample plot taken on a conductor pipe at platform Gail at a depth of 9 m show the dramatic change from a barnacle dominated assemblage in 2001 to one dominated by *Watersipora* in 2013 (Fig 6). Thus, the modeled high potential connectivity among the southeastern platforms along with high local retention of larvae on colonized platforms suggests that *Watersipora* is likely to remain colonized on these platforms into the future.

Second, the modeling predicts little to no potential connectivity between the four southeastern platforms, Grace, Gilda, Gail, and Gina, and the three northwestern platforms, Holly, Houchin, and Hogan. *Watersipora* was not detected on the three northwestern platforms during the 2001 or 2013 field surveys, despite the expansion in distribution of *Watersipora* among the southeastern platforms during this period. These results suggest that colonization of the three northwestern platforms by *Watersipora* is unlikely to occur via larval dispersal from the four southeastern platforms.

Third, the harbors showed little to no potential connectivity with any of the platforms with the exception of platform Gina. When interpreting the modeled potential connectivity from the harbors, it is important to consider a key assumption. Since the model has a 1-km horizontal grid, the small-scale hydrodynamics of the nearshore, driven by variations in bathymetry, shoreline topography, and other factors, are not included in the model. Thus the coastal flows used to model particle dispersal from harbors are higher and less variable than real nearshore flows. Consequently, the modeling estimates of dispersal from the harbors are likely overestimated. Thus for six of the platforms in the study, Grace, Gilda, Gail, Holly, Houchin, and Hogan, there is likely no potential connectivity with any harbors. Given the uncertainties of modeling the nearshore, the potential spread of *Watersipora* to platform Gina from the harbors may also be overestimated.

The limited potential connectivity of *Watersipora* between platforms in the southeast relative to those in the northwest and between platforms and harbors may have implications for the genetic structure of these populations that can be evaluated in future work. For example, genetic differentiation in coral species was evident between populations in the Flower Garden Banks reefs and colonies on offshore oil and gas platforms in the northern Gulf of Mexico [53, 54]. Mackie et al. [35] observed genetic differentiation in *Watersipora* along the California coastline, including differences between samples collected in two of our study harbors, Port Hueneme and Channel Islands. Because genetic markers are sensitive to exchange between populations [55], variation in the genetic structure of *Watersipora* between platforms and harbors could further support our conclusions regarding connectivity among habitats developed using the biophysical model.

### Nearshore vs. Offshore Dispersal

Shanks [22] compiled empirical data on the relationship between PLD and dispersal distance for 67 species and found that species with PLDs of less than one day had dispersal distances on the order of meters to 100s of meters. This relationship was also observed by Siegel et al. [23] using genetic estimates of dispersal distance for 32 species. These observed dispersal distances are much less than the modeled dispersal distances from the four southeasterly platforms in our study, which range from 1.1–1.4 km at a PLD of 3 hours to 9.6–11.5 km at a PLD of 24 hours (Fig 5). These modeled dispersal distances are supported by the 2001 and 2013 surveys, which observed the potential spread of *Watersipora* between platforms that are 5–10 km apart.

The hydrodynamic environment has been identified as an important driver of larval dispersal [2, 22, 56]. Due to shallow water and variable bathymetry, nearshore flows are slower and more complex than offshore flows. Shanks [22] hypothesized that species with short PLDs may only disperse a short distance in the nearshore simply because they are exposed to slow flows during their brief planktonic stage. Our results suggest that the enhanced dispersal of larvae with short PLDs released from offshore structures is driven, at least in part, by the high, sustained flows of the offshore hydrodynamic environment. Our modeling results show that *Watersipora* larvae could potentially travel up to10 km to a potential settlement site within 24 hours. However, the actual distance traveled is likely reduced by high mortality rates [57, 58] and by low post-settlement survival and growth due to delayed settlement [59–61].

Shanks [22] also hypothesized that organisms with PLDs of 12 hours or less may have short dispersal distances because they exhibit behavior that allows them to remain close to the sea floor, increasing their likelihood of encountering suitable habitat. This behavior is unlikely for *Watersipora* colonizing offshore platforms for two reasons. First, *Watersipora* was found in the top 18 m of the water column on the platforms during the 2001 and 2013 field surveys. This release depth is well above the seafloor as the four platforms where *Watersipora* was found are located in water depths ranging from 29 to 225 m. Second, typical of other bryozoan larvae, *Watersipora* initially show positive phototaxis on release (personal observation and [50]), which would result in larvae entering the water column. Unless these larvae encounter another part of the platform, this behavior increases the chance that larvae are potentially advected away from the platform by currents. Larval settlement would then depend on a chance encounter with another platform or being transported inshore to suitable habitat.

In conclusion, we hypothesize that the dispersal of *Watersipora* larvae and likely the larvae of other organisms with short PLDs, such as other bryozoans and ascidians [58], is greater when released in the offshore above the seafloor than when released in the nearshore and that the enhanced dispersal is driven by the high sustained flows of the offshore hydrodynamic environment. Our results further suggest that offshore habitat in general, such as pinnacles, shallow seamounts, and wind farms in addition to oil and gas platforms, could facilitate wider dispersal by sessile invertebrates with short PLDs. Therefore if connectivity between offshore structures is to be minimized, the distance between structures and the hydrodynamic environment should be considered.

## Supporting Information

S1 Table Unpublished data from settlement plates at platform Gilda. Mean number of *Watersipora* colonies on 15 x 15 cm ceramic tile settlement plates deployed and retrieved every three months at platform Gilda from June 2001 through May 2002. Mean number of colonies 1 ±SE, n = 4 plates.
(PDF)
