# Supplementary material for: The Effects of Anthropogenic Structures on Habitat Connectivity and the Potential Spread of Non-Native Invertebrate Species in the Offshore Environment
Source: PLoS One. 2016 Mar 31;11(3):e0152261. doi: 10.1371/journal.pone.0152261 (PMC4816305; doi:10.1371/journal.pone.0152261)
Supplement: S1 Table — Mean number of Watersipora colonies on 15 x 15 cm ceramic tile settlement plates deployed and retrieved every three months at platform Gilda from June 2001 through May 2002. Mean number of colonies 1 ±SE, n = 4 plates. (PDF) [file pone.0152261.s001.pdf]

Supporting Information

S1 Table. Unpublished data from settlement plates at platform Gilda. Mean number of *Watersipora* colonies on 15 x 15 cm ceramic tile settlement plates deployed and retrieved every three months at platform Gilda from June 2001 through May 2002. Mean number of colonies 1 ±SE, n = 4 plates.

**Date**	**No. *Watersipora* colonies**
June - August 2001	28 ± 7
September - November 2001	3 ± 2
December 2001 - February 2002	0
March - May 2002	0
